# Reintroducing vacuum extraction in primary health care facilities: a case study from Tanzania

**DOI:** 10.1186/s12884-018-1888-9

**Published:** 2018-06-19

**Authors:** Sunday Dominico, Patricia E. Bailey, Nguke Mwakatundu, Mkambu Kasanga, Jos van Roosmalen

**Affiliations:** 1 Thamini Uhai, Kigoma, Tanzania; 2 Pittsboro, USA; 3 Department of Obstetrics, Leiden University Medical Center and Athena Institute, VU University, de Boelelaan 1105, 1081 HV Amsterdam, The Netherlands

**Keywords:** Vacuum extraction, Caesarean delivery, Task-shifting

## Abstract

**Background:**

In rural Tanzania access to emergency obstetric and newborn care is threatened by poor roads and understaffed facilities among other challenges. Districts in Kigoma, Pwani and Morogoro regions were targeted by a local non-governmental organization to assist local government to build capacity and improve access to clinical management of severe obstetric and newborn complications. The program upgraded ten primary health care centres to provide comprehensive emergency obstetric and newborn care. This paper describes the process of reintroducing vacuum extraction into ten health centres and five hospitals, highlighting patterns in uptake, mode of delivery and lessons learned.

**Methods:**

This observational study uses facility-based trend data collected between 2011 and 2016.Descriptive outcomes include institutional caesarean delivery rates, vacuum extraction rates, and the ratio of caesareans to vacuum-assisted deliveries.

**Results:**

Institutional caesarean delivery rates remained stable at about 10–11% and the vacuum extraction rate rose from virtually no procedures in 2011 to about 2% in 2016. The increase was more visible in upgraded health centres than in hospitals. In 2016 vacuum extraction rates in newly upgraded health centres ranged from 0.5 to 7.8%. Between 2011 and 2016, the ratio of caesareans to vacuum extractions in hospitals changed from 304 caesareans to 1 vacuum extraction to 10:1, while in health centres the ratio changed from 22: 1 to 3: 1.

**Conclusions:**

Reintroduction of vacuum extraction into clinical practice in primary health care facilities with task-shifting is feasible. Reintroduction of this procedure was more successful when part of an integrated upgrading of health centres to provide comprehensive emergency obstetric care than when reintroduced into busy hospital environments. Turnover of trained staff in hospitals contributed to the uneven uptake of vacuum extraction. Lessons learned are applicable to further national scale up and to other countries.

## Background

Timely delivery of a newborn in the second stage of labour can mean the difference between life and death in childbirth for some women and their babies. Prolonged labour, a frequent complication, especially among nulliparous women [1], or suspicion of foetal distress, often leads to the need for rapid intervention. When labour is well monitored and indications align, assisting delivery with vacuum extraction (VE) can be successful in a short time. Taking the woman to an operating theatre for caesarean delivery, however, is the more frequent path of action, but one that takes longer, is costlier, and leaves the woman at high risk of serious complications in the index and future pregnancies.

Assisted vaginal delivery has slipped out of favour in many countries over the last few decades [2], but it has never been widely used in most low- and middle-income countries despite high rates of use in Western Europe, Canada and Australia. As caesarean rates rise, instrumental delivery is seen as one strategy to temper that rise, especially by preventing first caesareans among singleton term nulliparous women with spontaneous or induced labour [3]. In fact, VE is safer than surgery for the woman and her baby experiencing prolonged second stage of labour (unless signs of cephalopelvic disproportion are clear) [4]. Nevertheless, caesarean deliveries save thousands of lives each year and must be available when medically indicated, but a non-surgical option for women should also be encouraged when appropriate.

The socio-economic context for women with prolonged labour or foetal distress helps to determine what actions are taken. In Tanzania, a country with high levels of maternal and neonatal mortality (398 maternal deaths per 100,000 births, and 34 neonatal deaths/1000 live births, respectively) [5], the option of either procedure is undermined by lack of medical infrastructure, limited human resources, lack of training and equipment, and a fragmented referral system. Poor roads and limited transport options contribute to the difficulties labouring women face. According to the Tanzania Demographic Health Survey (DHS) 2015–16, the top two barriers to care-seeking were lack of money and distance to health facilities, two factors entwined [6]. Despite strong policy support from the government for institutional childbirth and a substantial investment in the primary health care system, for these and other reasons, over one-third of all births still occur at home without a skilled attendant at birth [6].

In 2008,^1^ the government of Tanzania supported by the World Lung Foundation embarked on a project known as Thamini Uhai (Swahili for ‘Value Life’) to improve access to, availability, and quality of comprehensive emergency obstetric and newborn care (C-EmONC) in 15 facilities in three regions (Kigoma, Morogoro and Pwani). The C-EmONC project started with two innovative core strategies for achieving its goals: 1) decentralization of life-saving services from district or regional hospitals to primary health care centres, and 2) task-shifting obstetric procedures from doctors, who are in short supply, to advanced level associate clinicians or assistant medical officers (AMOs), and anaesthesia to associate clinicians or nurse anaesthetists and clinical officers. Nine remote rural primary health centres and one urban health centre were upgraded to provide C-EmONC, i.e. they were staffed and equipped to provide obstetric surgery, blood transfusion as well as the basic EmONC signal functions [7]. The strategy of expanding the role of selected health centres was strongly embraced by Tanzania’s Ministry of Health that included decentralization and task-shifting as leading interventions in the nation’s health policies and strategies to reduce maternal and perinatal mortality.

Five district or regional hospitals that received referrals from the ten health centres also benefitted from project quality improvement aspects (human resource support, training, clinical audit, on-going mentoring supervision). The project has demonstrated that traditional and innovative strategies were not only feasible but they also substantially increased institutional childbirth rates, access to life-saving services, and the quality of care. Furthermore, they decreased the need for referral [8, 9].

This paper describes a lesser recognized aspect of Thamini Uhai’s implementation success, namely how they reintroduced VE into mainstream clinical practice. It also describes patterns in the uptake of VE, trends in mode of delivery, and lessons learned in the process.

## Methods

### The VE reintroduction process

Assisted vaginal delivery in Tanzania had been all but abandoned in 2008 when Thamini Uhai began implementation. Obstetric forceps was never popular and although national guidelines recognized VE as a sanctioned obstetric procedure, its utilization was often not considered [10, 11]. Quality improvement audits in two Tanzanian hospitals suggested that fear of HIV contributed to the reluctance to use VE [11, 12].^2^ A guiding principle to capacity building was that all associate clinicians who were going to engage in surgery also needed the skills to use a vacuum extractor.

Before implementing VE training at project sites, advocacy efforts strategically began with clinical training at the two largest medical training centres in the country: Muhimbili Medical School and Bugando Teaching Hospital, where the country’s leading obstetrician/gynaecologists worked. International experts teamed up with local champions to conduct these trainings. Some participants expressed reservations while paediatricians in some regions, at least, spoke against the practice. Nevertheless, the outcome of this early attempt to elicit the support of local leaders in the field was one of strong endorsement by the Ministry of Health and it fostered a favourable climate for including VE in the toolbox of obstetric skills.

The task-shifting strategy that Thamini Uhai embraced builds on a long history in Tanzania of working with AMOs for surgery [13] and nurse-midwives and clinical officers for anaesthesia. The 3 months of EmONC training for teams of surgeons and anaesthetists has been described elsewhere [14]. It also included skills-building in VE, removal of retained products, manual removal of placenta, cervical and perineal repairs, and adult and neonatal resuscitation.

This training was followed by weeklong Continuing Medical Education (CME) sessions that focused on specific topics that were identified as requiring further confidence- and skills-building such as VE. CMEs were conducted at the five hospitals in the three regions. Two rounds of VE CMEs have taken place, one in 2012 and the other in 2016. The CME focused on a review of the partograph and VE. Trainees used anatomical models for practice but had relatively few opportunities themselves to perform a vacuum-assisted delivery on a patient, but all observed the procedure being performed. Between nine and 14 individuals made up a batch for a CME session. The training used both soft and metal cups and the project distributed Malmström extractors and Kiwi equipment. The training was considered competency-based in the context of anatomical models and followed a protocol adapted from the American College of Obstetricians and Gynecologists. Training in VE also included how to treat complications such as postpartum haemorrhage and perineal lacerations, if they occurred. Because all sites had an operating theatre (OT), if vacuum-assisted delivery was not successful, a woman was transferred to the OT for caesarean delivery.

Multiple activities were put in place to support the newly trained AMOs, nurse-midwives and clinical officers, and to improve the quality of care. These included supportive supervision and clinical audits. Obstetrician/gynaecologists or experienced AMOs and an expert anaesthetist visited each site monthly, spending two to three-days at each site, providing on-the-job coaching and hands-on mentoring. The project established weekly teleconferences allowing staff to discuss specific cases with clinicians as well as closed user group networks enabling each facility to make calls for free within the network. The closed user groups facilitated specialists to be ‘on call’ so that emergency consultations were possible day and night; in Kigoma, they created a “WhatsApp” group.

The supportive supervision and clinical audit visits were helpful in identifying when a CME was warranted as staff rotated and others were transferred. In addition to the hands-on CMEs, eLearning sessions were developed. In the case of VE, the trainers adopted WHO’s video on VE and translated it into Swahili.

The supervisory teams also reviewed the contents of a monthly monitoring form that tracked aggregated service statistics – births, maternal and neonatal deaths, intrapartum stillbirths, near misses, and audits of all caesareans, which were used for quality improvement purposes and immediate feedback [8]. The data extracted from these forms were used in the analyses below. When retrieving this information, staff had no contact with patients and no names were captured on the form. Given that the primary purpose of the data was for internal quality improvement and feedback for the staff and to document high level changes over time, neither patient consent nor Institutional Review Board approval was sought. However, the Ministry of Health and Social Welfare, the Regional and District health management teams and medical directors of participating facilities granted permission and approval of project activities.

In 2015 four additional health centres were selected for C-EmONC upgrading in Kigoma and began receiving a similar package of interventions that the other sites received, including training in VE for AMOs and nurse-midwives.

For this paper, the monitoring counts were used to calculate percentages, rates and ratios, for example, the ratio of the number of caesareans to VE procedures. No statistical tests were used.

## Results

Anaesthesia and caesarean delivery skills-building was a central thrust of upgrading health centres between 2008 and 2011. Although VE was included, the first intensively focused CME on VE was conducted in 2012. Over the course of the project, at least four persons were trained in the use of VE at each hospital, and two at each health centre. At last count, more than 80 providers completed the three-months C-EmONC course and were trained to perform VE; 56 providers received CME training in 2016 alone. Initially, most VE trainees were AMOs, who were called only when midwives encountered a difficult delivery or complications. In the last Continuous Medical Education workshop, 75% of trainees were nurse-midwives purposefully.

As a backdrop to the uptake and performance of emergency obstetric procedures, the annual number of women giving birth in the project facilities increased by 26% at the five hospitals, from 10,950 deliveries to 13,810, between 2011 and 2016 (Table 1). The increase at the ten health centres was 22%, from 10,788 deliveries to 13,152, for an overall increase of 24% (Table 1). The increase was not evenly spread across project sites; it was highest (30%) in Pwani region (two project sites), next highest (28%) in Morogoro (four sites) and lowest (17%) in Kigoma (nine sites).

**Table 1:** Deliveries in detail in the original 15 project supported facilities (2011–2016)

Years	2011	2012	2013	2014	2015	2016
Hospitals (*n* = 5)						
Total deliveries	10,950	12,056	12,376	13,729	13,910	13,810
Caesarean deliveries	1822	1858	1609	1901	1857	2163
Vacuum extractions	6	71	185	115	133	219
Health centres (*n* = 10)						
Total deliveries	10,788	11,436	10,861	12,299	12,279	13,152
Caesarean deliveries	624	692	580	670	709	852
Vacuum extractions	28	136	307	285	198	247
All facilities (*n* = 15)						
Total deliveries	21,738	23,492	23,237	26,028	26,189	26,962
Caesarean deliveries	2446	2550	2189	2571	2566	3015
Vacuum extractions	34	207	492	400	331	466

**Fig. 1:**
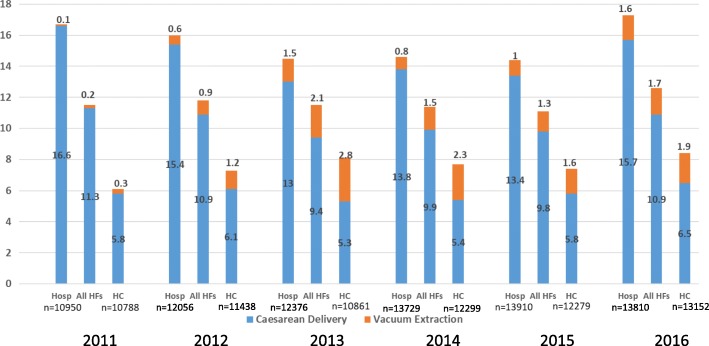
Trends in caesarean delivery and vacuum extraction rates (per 100 deliveries) among the 15 original project facilities

In Fig. 1, the total *assisted delivery rate*, defined as the proportion of women requiring an intervention for delivery, either abdominal or vaginal [15], ranged from 11.5% in 2011 to 12.6% in 2016, not much change overall. The proportion of deliveries by caesarean ranged from 9.4 to 11.3% and of VE deliveries 0.2 to 2.8%. The year 2013, following the VE CME training in 2012, showed the lowest caesarean rate and the highest VE rate, suggesting that the uptake of VE marginally might have replaced a few caesarean deliveries. Mentoring and supervision might have played a role in building confidence and encouraging the use of VE.

The upgrading of health centres to C-EmONC centres was a departure from the conventional division of services within the national health system. Hospitals provided approximately twice the “assisted delivery” interventions to their patient mix than did health centres (Fig. 1). From an external source of data, the*2016 Pregnancy Outcomes Study in Kigoma Region*, the investigators observed that the Kigoma hospitals received proportionately about twice as many complications as did health centres [16]. The incorporation of VE into providers’ daily practice was reportedly easier at health centres than hospitals and Fig. 1 supports this observation. Beginning in 2012, proportionately more deliveries were assisted with VE in health centres than in hospitals.

A more nuanced way of showing the variation in provider practice between hospitals and health centres is the ratio of caesarean deliveries to vacuum extractions (Table 2). In 2011 among hospitals, for every VE performed, clinicians and AMOs conducted 304 caesareans. In 2016 this ratio was 10 caesareans to 1 VE. In health centres, we see the same direction of change but at a different magnitude: from 22 caesareans to 1 VE in 2011 to a ratio of 3: 1 in 2016.

**Table 2:** Trends in the ratio of caesarean deliveries to vacuum extractions in hospitals and health centres

	2011	2012	2013	2014	2015	2016
Hospitals	303.7	26.2	8.7	16.5	14.0	9.9
Health centres	22.3	5.1	1.9	2.4	3.6	3.4

**Fig. 2:**
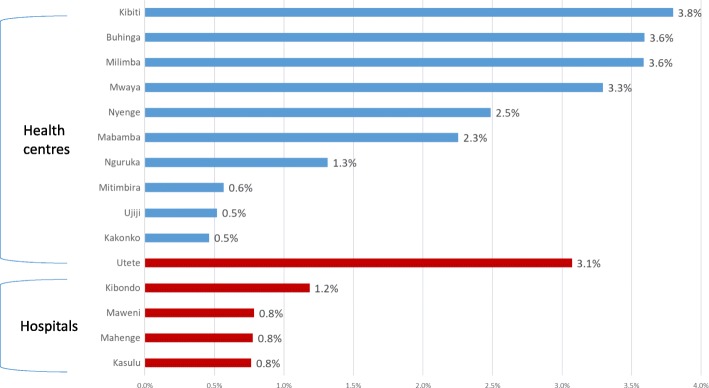
Proportion of vacuum-assisted deliveries in supported sites 2011–2016

The aggregation of facilities shows overall trends but masks provider practice variation at health facility level. The disaggregation of facilities (Fig. 2) into health centres and hospitals reveals an uneven pattern in uptake and raises the question why uptake was greater in some facilities than in others. In these figures, the deliveries and VEs performed between 2011 and 2016 were reported. Four health centres had a VE rate of greater than 3% while three had a rate of less than 1%. The hospital of Utete was the only hospital with a VE rate of 3.1% while less than 1% of deliveries were delivered by VE in three of the five hospitals.

**Fig. 3:**
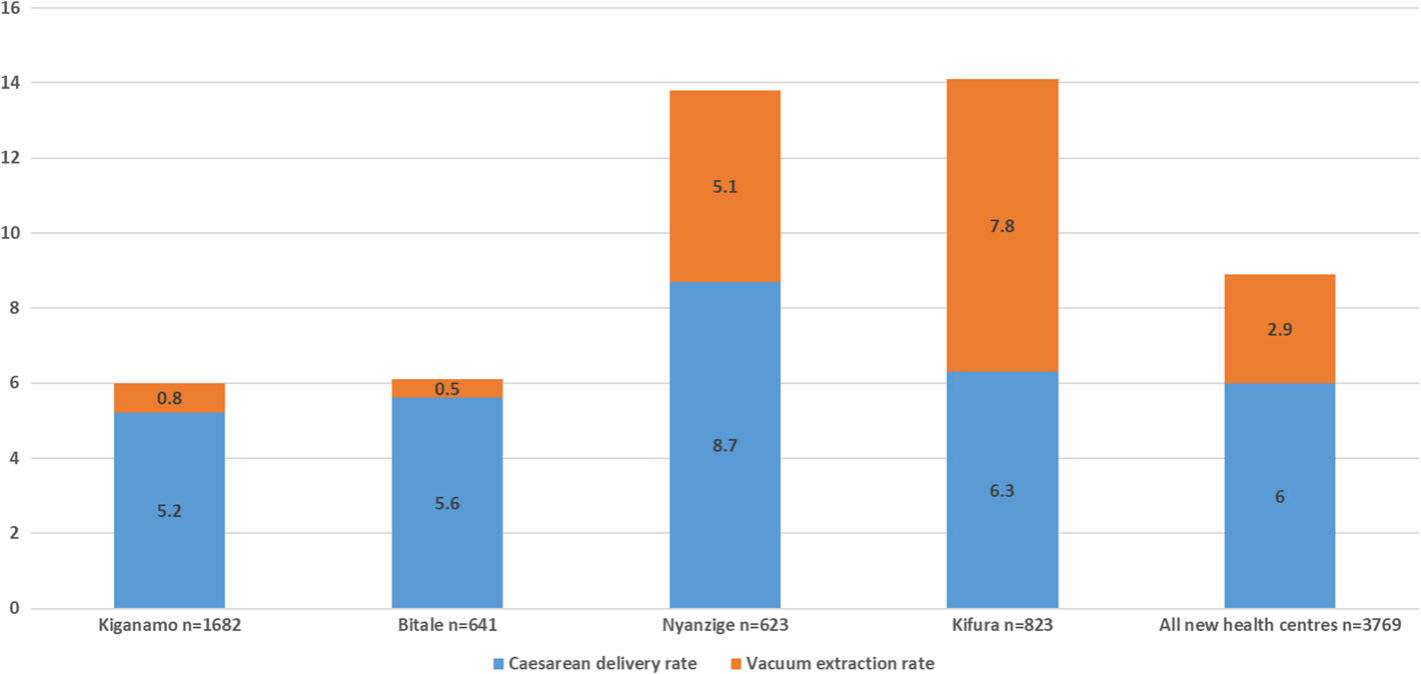
Proportion of deliveries by caesarean and vacuum extraction in 2016 among health centres added in 2015

Based on data from the four health centres in Kigoma that were added to Thamini Uhai’s scope of support in 2015, we see a similar uneven pattern of the uptake in VE in Fig. 3. At Kifura and Nyanzige health centres 7.8 and 5.1% of deliveries, respectively, were assisted with VE while less than 1% of deliveries at the other two health centres involved vacuum assistance. In fact, at Kifura, marginally more deliveries were assisted by VE than delivered by surgery.

### Impact on intrapartum stillbirths

Improved emergency obstetric care should bring about improved perinatal outcomes, specifically fewer intrapartum stillbirths and early neonatal deaths. The *2016 Pregnancy Outcomes Study in Kigoma Region*, carried out by the USA Centers for Disease Control and Prevention, indicated a decline in the overall institutional stillbirth rate (from 28/1000 births to 22/1000 births) as well as the intrapartum stillbirth rate (from 16/1000 births to 12/1000 births) between 2011 and 2015, respectively. The highest rates were identified in project-supported hospitals but these facilities also showed the greatest declines (from 37 to 20/1000 births). Project-supported health centres showed a small increase (from 12 to 16/1000 births) [16]. Unfortunately, no similar data exist for project sites in Morogoro or Pwani.

## Discussion

This paper describes an experience reintroducing vacuum extraction into a context where it was rarely practiced. Vacuum extraction was reintroduced not as a reaction to rising costs due to soaring caesarean delivery rates as it has been in several Latin American hospitals [17, 18], but motivated by providing evidence-based care and preventing highly interventionist strategies that sometimes lose focus of a woman’s well-being, the physical and mental hardships related to recovery from surgery, future pregnancy risks, and the financial costs to families and the health system.

In this case, VE has become part of an expanded skill set aimed at providing care to pregnant women who live in rural and often remote areas. Nyamtema and co-authors have described many successes of Thamini Uhai – the increase in institutional childbirth [8], effective task-shifting [14], and improved quality of care measured by a decrease in unjustified caesarean deliveries, and a lower risk of dying from complications of caesarean delivery [9]. VE has played a role in this changing environment since a non-surgical option now exists for selected cases of foetal distress and prolonged second stage of labour. Surely, some caesarean deliveries have been averted and intrapartum stillbirth rates are on the decline, although the introduction of VE is likely to be just one of several contributing factors.

Caesarean rates in the three regions that Thamini Uhai has supported are not excessive. According to the 2015–16 DHS and Multiple Indicator Survey, the population-based caesarean rates in Kigoma, Morogoro, and Pwani were 4.0, 6.2, and 3.0%, respectively [6]. In this paper, we saw rates of VE ranging from less than 1 to 7.8% among facility-based deliveries. The ratio of caesarean deliveries to VE, however, changed dramatically over the 6 years that Thamini Uhai collected mode of delivery data. By 2016 hospitals were intervening at a 10: 1 caesarean to VE ratio while upgraded health centres intervened at a 3: 1 ratio. To put these in a global context, the ratio in Scotland, Ireland, Canada, Australia and England (between 2004 and 2009) hovered around 2 to 1, and in the United States 7 to 1 [15]. In several sub-Saharan African countries, however, there is a tendency towards a higher dependency on caesarean surgery, for example, 27 caesareans to 1 VE in Congo Brazzaville in 2012 and 22: 1 in Ghana in 2010 [2].

This experience points to several lessons learned and challenges when reintroducing an underutilized clinical practice. It took the 2012 CME session dedicated to VE to jumpstart its integration into practice and another in 2016 to reinvigorate its use. Frequent staff turnover meant that training and coaching became an on-going need. This is not unique to VE but a universal complaint of training [19–21]; too often a person with a newly desirable skills-mix is transferred to a non-supportive or unequipped environment and skills are lost. Equipment is often cited as a barrier to performing VE [2], but it has not been a major issue in the context of the Thamini Uhai project environment. Yet maintenance and the acquisition of new equipment could become a challenge as the government takes greater responsibility of the project sites. To our knowledge, adverse outcomes related to VE have not produced a setback. Each hospital and health centre where VE is practiced has had caesarean surgery as a backup. That said, based on the monitoring of birth outcomes, the project has yet to show a definitive decrease in the intrapartum stillbirth rate at all levels, unlike a recent study in a well-known university hospital in Uganda that showed a strong association between VE reintroduction and fewer intrapartum stillbirths [22]. The lack of progress on this parameter has concerned staff; efforts to improve referral, audits of newborn deaths, training in neonatal resuscitation, and careful monitoring of newborn outcomes at health centres are underway.

The observation of the uneven uptake of VE across facilities is important for scaling up VE or for others attempting to reintroduce VE into an established practice. Major differences in training were ruled out as an explanation since the trainees from different facilities were brought together and trained by the same trainers using the same methodology. Several facilities – Utete, Buhingu, Nyenge, Mabamba and Mwaya – enjoyed more favourable ratios of health workers to delivery volume than other facilities, which might have helped explain their success of mainstreaming VE. The difficulty of monitoring labour that adheres to standards is not surprising where there is a shortage of staff. What the facilities with the highest VE rates seem to have had in common were AMOs and nurse-midwives that stood out for their commitment and activity level. Where the uptake of VE was particularly slow, additional staff members were trained, hoping that would provide a solution. Yet the procedure mix has not changed, suggesting that there may be pockets of resistance to the use of VE specifically, or resistance to changing practices more generally.

The low VE practice at supported hospitals could partially be explained by high staff-turnover as there have been staff rotations at least annually, and these have been replaced with others possibly without the skills or interest. The number of staff trained in VE does not seem to be the significant factor, since during the last phase of training between two and four providers were trained at each of the supported health centres and hospitals. High turnover of staff, however, is probably not the only explanation for low VE usage or for high reliance on caesarean delivery in hospitals. We also know that hospitals attended twice the number of obstetric complications than health centres did, and a large proportion of these were referrals of women with more serious conditions and with a wider range of indications for surgery.

The relatively rapid uptake of VE by the newly renovated health centres compared with hospitals where caesarean delivery was well entrenched may not come as a surprise for those who study the phenomenon of early adoption. The personal risk of using a new skill in a recently enhanced environment where the clinical staff was trained together may be lower than in facilities with more staff, not all of whom received the same training, and where clinical practices may be harder to change. It has been noted that the project stimulated and motivated staff, giving them a greater sense of satisfaction and confidence by enabling them to provide more services in improved conditions. Providing feedback to the facilities themselves on the wide variations in VE practice and adoption, much like an audit cycle, may also lead to internal discussion and reflection.

As Thamini Uhai’s support shifts towards working less on the front line to more support to government structures to ensure the sustainability of what has been achieved, continued use of VE may be tested. Local policy support will be needed to keep unnecessary medical interventions at a minimum while championing the nurse-midwives, AMOs and specialists who currently mentor and train new staff arrivals.

Strengths of this case study include the project’s ability 1) to monitor facility service statistics for at least 6 years by a stable implementation support team using the same methodology, 2) to observe not just long-term changes but short-term changes in sites that were added in 2015, and 3) to compare hospitals with health centres that have undergone substantial transition to fully functioning C-EmONC centres. A limitation, as implied above, is a lack of firm understanding of why staffs at some facilities perform VE more than others. Other gaps in information included indications for VE and caesarean delivery as well as any adverse outcomes related to each procedure, all of which would enrich the policy and program debate in Tanzania and elsewhere. Although not systematically recorded, serious complications resulting from vacuum extraction would have been revealed during project expert teams’ monthly and quarterly supportive supervision and mentorship visits to the facilities. Finally, like many multi-faceted programmatic interventions, without a more rigorous evaluation design, attribution of cause and effect may be driven more by plausibility than definitive proof.

The maturity of the program should facilitate initiating an opportunity to qualitatively explore whether newly trained staff lack confidence, if physical or political environments are not supportive and how training and coaching might be modified. Simply listening and learning from both the slow and the early adopters, in both hospitals and health centres will provide much needed insight to support further efforts to reintroduce VE. Continuing to document this long term, multi-site experience to show safety of VE, cost savings and improvements in outcomes, will not only help local scale up but also influence national policy and provide guidance to others.

## Conclusions

Although reintroduction of vacuum extraction is feasible, it requires a supportive environment that is sensitive and follow-up to when refresher training is needed. In this Tanzanian context, it seems to have been easier to reintroduce vacuum extraction as part of an integrated upgrading of health centres to provide C-EmONC than it has been to introduce it into a busy hospital environment.

## Abbreviations

AMO: Assistant medical officer
C-EmONC: Comprehensive emergency obstetric and newborn care
CME: Continuing medical education
DHS: Demographic and Health Survey
Ob/gyn: Obstetrician/gynaecologist
OT: Operating theatre
VE: Vacuum extraction

## Footnotes

1 In 2006 groundwork for the project started but it was not formally supported by Bloomberg Philanthropies or the World Lung Foundation until 2008.

2 The 2017 edition of *Managing complications in pregnancy and childbirth: a guide for midwives and doctors* recommends that artificial rupture of membranes not be the sole intervention for labour augmentation in areas of high HIV prevalence, but the authors do not caution against vacuum-assisted delivery in these same settings.
